# Proteomic Assessment of Extracellular Vesicles from Canine Tissue Explants as a Pipeline to Identify Molecular Targets in Osteosarcoma: PSMD14/Rpn11 as a Proof of Principle

**DOI:** 10.3390/ijms23063256

**Published:** 2022-03-17

**Authors:** Anita K. Luu, Mia Cadieux, Mackenzie Wong, Rachel Macdonald, Robert Jones, Dongsic Choi, Michelle Oblak, Brigitte Brisson, Scott Sauer, James Chafitz, David Warshawsky, Geoffrey A. Wood, Alicia M. Viloria-Petit

**Affiliations:** 1 Department of Biomedical Sciences, Ontario Veterinary College, University of Guelph, Guelph, ON N1G 2W1, Canada; aluu@uoguelph.ca (A.K.L.); mcadieux@uoguelph.ca (M.C.); mackenzie.wong@hc-sc.gc.ca (M.W.); rachel.macdonald@mail.utoronto.ca (R.M.); 2 Department of Animal Biosciences, Ontario Agricultural College, University of Guelph, Guelph, ON N1G 2W1, Canada; rjones12@uoguelph.ca; 3 Department of Biochemistry, College of Medicine, Soonchunhyang University, Cheonan 31151, Korea; dongsicchoi@gmail.com; 4 Department of Clinical Studies, Ontario Veterinary College, University of Guelph, Guelph, ON N1G 2W1, Canada; moblak@uoguelph.ca (M.O.); bbrisson@uoguelph.ca (B.B.); 5 Vuja De Sciences, Inc., Natick, MA 01760, USA; scott.sauer@vujade-life.com (S.S.); dw@vujade-life.com (D.W.); 6 SBH Sciences, Natick, MA 01760, USA; jchafitz@sbhsciences.com; 7 Department of Pathobiology, Ontario Veterinary College, University of Guelph, Guelph, ON N1G 2W1, Canada; gewood@uoguelph.ca

**Keywords:** osteosarcoma, extracellular vesicles, tissue explants, biomarker discovery, molecular targets

## Abstract

Osteosarcoma (OS) is a highly malignant bone tumour that has seen little improvement in treatment modalities in the past 30 years. Understanding what molecules contribute to OS biology could aid in the discovery of novel therapies. Extracellular vesicles (EVs) serve as a mode of cell-to-cell communication and have the potential to uncover novel protein signatures. In our research, we developed a novel pipeline to isolate, characterize, and profile EVs from normal bone and osteosarcoma tissue explants from canine OS patients. Proteomic analysis of vesicle preparations revealed a protein signature related to protein metabolism. One molecule of interest, PSMD14/Rpn11, was explored further given its prognostic potential in human and canine OS, and its targetability with the drug capzimin. In vitro experiments demonstrated that capzimin induces apoptosis and reduces clonogenic survival, proliferation, and migration in two metastatic canine OS cell lines. Capzimin also reduces the viability of metastatic human OS cells cultured under 3D conditions that mimic the growth of OS cells at secondary sites. This unique pipeline can improve our understanding of OS biology and identify new prognostic markers and molecular targets for both canine and human OS patients.

## 1. Introduction

Osteosarcoma (OS) is an aggressive malignancy of the bone that has a high metastatic rate [1]. Although the incidence of OS is rare in humans, accounting for fewer than 1000 cases per year in the USA, the incidence is notably higher in dogs and, in 2007, was estimated to be more than 8000 dogs each year [2]. This is likely an underrepresentation as the incidence rate in canines has been suggested to be 27 times higher than that of humans [3,4]. In canines, it is well known that large and giant breeds have an increased risk of developing OS, with the median age of diagnosis being 7 years [2,5]. A majority of OS cases present in the metaphyseal region of long bones; however, cases can also present on cortical surfaces and, in rare instances, in extraskeletal locations [6,7]. Clinical manifestations of OS include pain, lameness, and swelling in the affected limb which become increasingly more severe [8]. Diagnostic work-up involves a radiograph of the affected limb which appears as a lytic or osteoblastic lesion. Radiographic findings, physical examination and clinical presentation are typically enough to suggest OS, but a histopathological assessment of a tissue biopsy provides a more definitive diagnosis [2]. It is important that OS patients also receive 3-view thoracic radiographs, typically involving right and left lateral and ventrodorsal or dorsoventral views, to detect the presence of pulmonary metastases [9]. Although it is predicted that 90% of canine patients have micrometastases at the time of diagnosis, less than 15% of OS patients have radiographically detectable lesions at initial presentation [2,10,11].

The current standard of care includes limb-spare or limb amputation, followed by chemotherapy. Typical adjuvant chemotherapeutics include doxorubicin, cisplatin, or carboplatin and have extended median survival to 8–12 months, depending on the agent [10,11,12,13,14,15,16,17,18,19,20]. This is a significant improvement compared to the reported median survival time of 4–6 months when patients are treated with surgery alone [12,13,15,16,20]. To date, no novel therapeutic advances have led to meaningful changes in survival outcomes for OS patients. This underscores the need to better understand OS biology, discover novel molecular targets, and find biomarkers that could assist in personalized medicine. Profiling extracellular vesicles (EVs) could be a powerful tool to advance all these aspects.

EVs represent a heterogenous population of membrane-bound vesicles that are released into the extracellular environment [21]. They are typically classified by their size and mode of secretion, and their contents include a diverse range of biomolecules including DNA, RNA, miRNA, protein, lipids, and metabolites. Exosomes are 40–120 nm in diameter and are formed by endocytosis of the plasma membrane and mature in multivesicular bodies. Microvesicles (sometimes referred to as ectosomes, microparticles, or shedding microvesicles) are 100–1000 nm in diameter and shed directly from the plasma membrane. Apoptotic bodies range from 50 nm to 4000 nm and result from fragmented apoptotic cells, and oncosomes are the largest subpopulation ranging from 1000–10,000 nm [22]. EVs serve as a mode of intercellular communication and can also fuse with target cells to deliver biological materials [23,24].

The capabilities of EVs are diverse and have been implicated in virtually all aspects of cancer development and progression. In OS, previous work has shown that exosomes can mediate chemoresistance through the transfer of multidrug resistance mRNA and P-glycoprotein, and aid in migration by acting as chemo attractors [25,26]. EVs released by OS cells can also modulate the behaviour of stromal cells, supporting the creation of a pro-tumour microenvironment. Exosomes from OS cells can also be immunosuppressive, as they can inhibit T cell proliferation and promote a M2 macrophage (tumour-promoting) phenotype [27]. Lastly, OS-derived exosomes induce a cancer-associated fibroblast phenotype in lung fibroblasts, aiding in the development of the pre-metastatic niche [28].

Another exciting avenue for EV research is their use for biomarker discovery. As EV composition reflects their progenitor cell, characterizing EV content can help enhance our understanding of overall tumour biology [29]. A large proteomic characterization of vesicle cargo from the NCI-60 cancer cell lines found that EV proteomes tend to naturally cluster with their cell type. There also were unique subclustering patterns observed, likely reflecting their intrinsic cell properties such as metastatic potential [30]. A similar study was completed in breast cancer cell lines which found that EV proteomes could sufficiently distinguish breast cancer cell lines by their molecular subtype as either triple negative or HER2-positive. These proteomes also revealed distinct upregulation of various cellular processes [31]. In vitro work has also expanded to include more clinically relevant samples. More recently, Hoshino and colleagues profiled EVs from lung adenocarcinoma and pancreatic adenocarcinoma tumour tissue and found distinct proteomic signatures that reflect unique cellular processes [32].

The EV proteome has not yet been well characterized in OS, representing a missed opportunity to expand our understanding of OS biology. To this end, we present a novel pipeline that allows for the isolation, characterization, and profiling of EVs from non-malignant and malignant bone tissue explants from canine appendicular OS patients to aid in biomarker and molecular discovery. Tissue explants were chosen because they have the ability to capture patient-to-patient variability and the complexity of in vivo conditions, further improving the translational potential of our work. Our data demonstrates that EVs can be isolated from explant cultures and lead to the identification of novel prognostic and molecular targets of potential benefit to both canine and human OS.

## 2. Results

### 2.1. Extracellular Vesicles Can Be Successfully Isolated from OS and Normal Bone Tissue Explants

**Figure 1:**
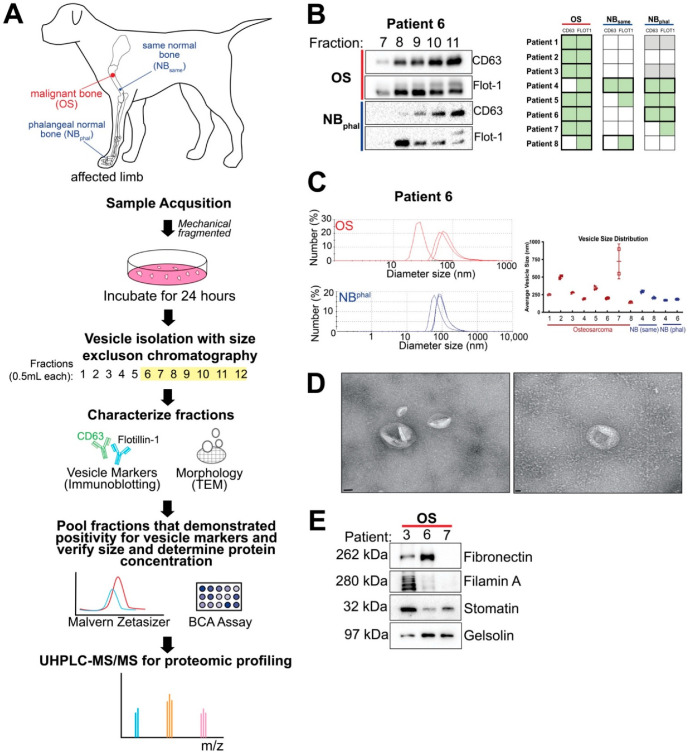
Isolation and characterization of extracellular vesicles from OS and non-malignant bone tissue explants. (**A**) General workflow for sample acquisition and vesicle characterization from canine appendicular OS patients. (**B**) Representative immunoblot for extracellular vesicle markers, CD63 and flotillin-1 (Flot-1) for patient 6 (left) and table summarizing immunoblot results for all patients (right). Green filled boxes represent positivity for the marker, empty boxes represent no signal detected for the marker, grey filled boxes indicate the sample was not obtained. Bold outlines represent the samples that were included in the UHPLC-MS/MS analysis as they were positive for CD63 and/or flotillin and had a total protein concentration of 2.9 μg (*n* = 12). (**C**) Representative size distribution analysis for pooled fractions that were CD63 and/or flotillin-1-positive for patient 6 (left) and summary graph of vesicle size distribution for all patients (right). (**D**) Representative transmission electron microscopy images of a vesicle preparation obtained with our protocol; scale bar = 50 nm (left) or 20 nm (right). (**E**) Immunoblots of OS samples from patients 3, 6, 7 for novel vesicle markers fibronectin, filamin A, stomatin and gelsolin.

In total, the owners of eight canine appendicular osteosarcoma patients undergoing limb amputation provided consent to take part in this study. Tissue samples were obtained immediately following surgical limb amputation. OS samples were acquired from the tumour site, while two non-malignant bone samples were taken: one from a different region on the same bone (NB_same_) and one sample of phalangeal bone (NB_phal_) to serve as controls. Samples were cultured for 24 h, and vesicles were isolated and characterized for vesicle marker expression, size, and protein concentration (see Figure 1A for workflow diagram). The first step to validate successful vesicle isolation was immunoblotting of isolated fractions for vesicle markers CD63 and flotillin-1 (see Figure 1B for representative immunoblot from a patient sample). Of the eight patient samples obtained, almost all vesicles isolated from OS samples were CD63 and flotillin-1-positive. However, this was only true for a small number of vesicle samples isolated from NB_same_ and NB_phal_ tissue (see table in Figure 1B). To determine the size of these vesicles, isolated fractions that were either CD63-positive, flotillin-positive, or CD63 and flotillin-positive, were pooled and subjected to dynamic light scattering (see Figure 1C, for representative analysis from a patient sample). The average vesicle size of OS samples was 312 nm ± 178 nm, NB_same_ was 246 nm ± 46.1 nm, and NB_phal_ was 177 nm ± 7.68 nm (Figure 1C, right). We also evaluated vesicle preparations isolated with this protocol using transmission electron microscopy and observed a morphology that is characteristic of vesicles (Figure 1D). To determine the proteomic profile of these vesicles, we completed ultra-high-performance liquid chromatography and tandem mass spectrometry (UHPLC-MS/MS) on the pooled fractions that demonstrated positivity for flotillin expression. Unfortunately, only two NB_same_ and two NB_phal_ vesicle preparations had an adequate protein concentration (2.9 μg) for accurate proteomic analysis. A total of 12 samples (8 OS, 2 NB_same_ and 2 NB_phal_) were subjected to UHPLC-MS/MS. In attempt to validate the proteomic data, immunoblotting was completed in three OS samples, which evaluated the expression of four novel putative vesicle markers [32]: fibronectin, filamin A, stomatin, and gelsolin (Figure 1E).

### 2.2. Twenty-Nine Proteins Are Significantly Increased in OS-Derived Vesicles Compared to Normal Bone-Derived Vesicles

**Figure 2:**
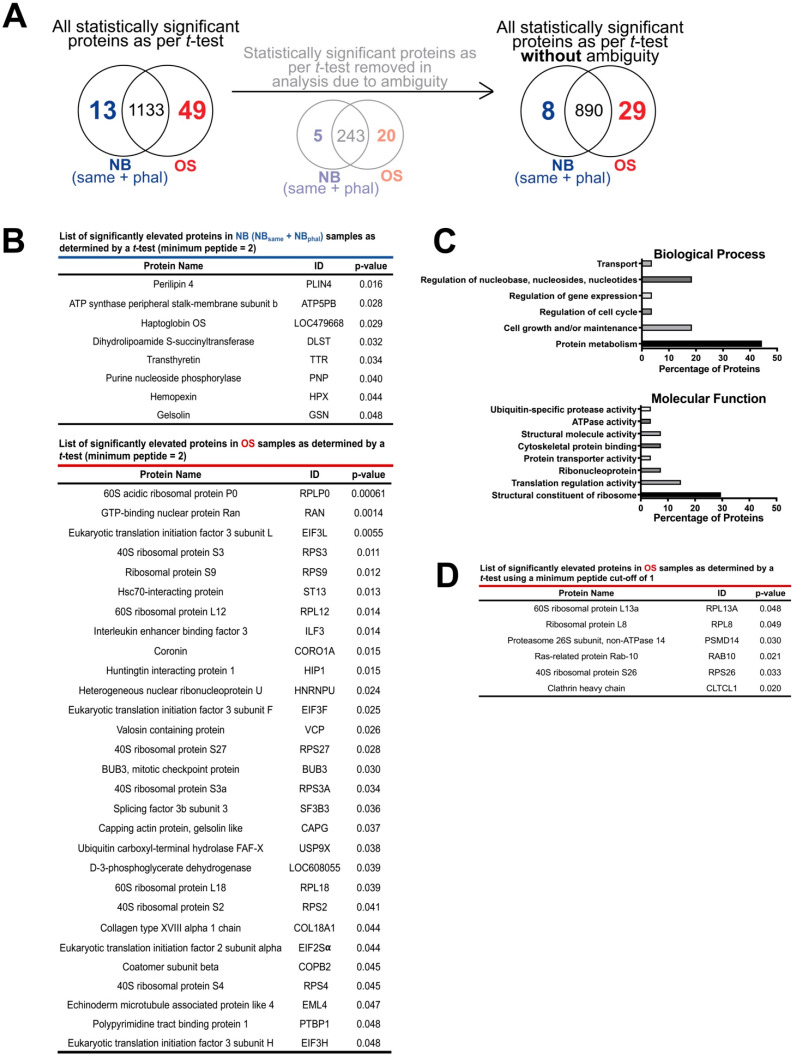
Proteins identified in UHPLC-MS/MS analysis including all samples. (**A**) Pie charts showing the number of statistically significantly proteins when comparing non-malignant bone (NB) and OS samples using a *t*-test. (**B**) Table of significantly elevated proteins found in the non-malignant bone samples (top) and OS samples (bottom) when using a minimum peptide = 2 as a cutoff and a *t*-test. (**C**) The molecular functions and biological processes of the significantly elevated proteins identified in the OS samples. (**D**) Table of significantly elevated proteins in OS samples when using a minimum peptide = 1 as a cutoff and a *t*-test.

As recommended for label-free quantification of proteins identified by mass spectrometry, we initially quantified proteins by normalized weighted spectra counting and used a *t*-test to compare OS (*n* = 8) and NB_same_ (*n* = 2) samples, as these were the ones that met the criteria of a similar number of identified protein and spectra. Using a protein and peptide threshold of 95% and a minimum of two peptides, we identified the proteasome subunit alpha type-7 (PSMA7) as significantly overexpressed in OS versus NB (*p* < 0.05). Given the small NB_same_ sample size, we decided to group NB_same_ and NB_phal_ together (*n* = 4) to collectively serve as a non-malignant control for the OS samples (*n* = 8) and therefore conduct an exploratory analysis to identify other proteins of interest. Using a protein and peptide threshold of 95% and a minimum of two peptides, we identified 927 proteins without ambiguity in our samples. Using a *t*-test, we found eight proteins which were significantly elevated in NB samples compared to OS, and 29 proteins which were significantly elevated in in OS compared to NB samples (Figure 2A,B). Of the proteins significantly elevated in the OS derived EV samples, almost half (45%) were proteins associated with protein metabolism. Further characterization of these proteins in terms of molecular function revealed that proteins associated with the structural component of the ribosome and translation regulation were most abundant (Figure 2C). A similar trend was observed for the proteins identified using a minimum peptide cut-off of 1 to compare OS vs. NB (Figure 2D). Of note, this identified six additional proteins, among them the proteasome 26S subunit, non-ATPase 14 (PSMD14), another proteasome component, as significantly upregulated in OS.

### 2.3. Proteasome Components Are Clinically Relevant and of Current Interest in the Field of OS

**Figure 3:**
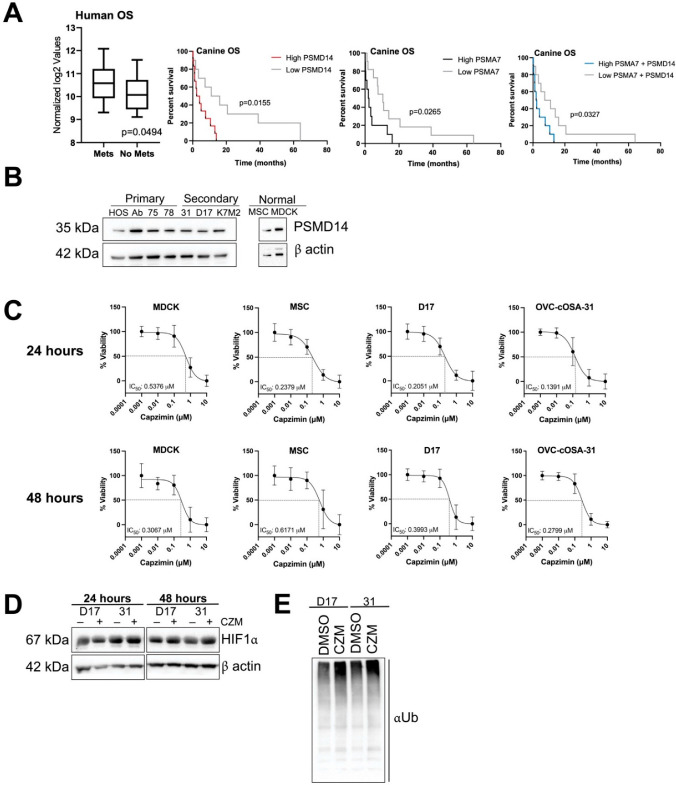
PSMD14 is a clinically relevant and viable target in OS. (**A**) Significant association between PSMD14 mRNA levels in treatment-naïve biopsies and the development of metastasis within 5 years (GSE21257, unpaired *t*-test). Significant difference in survival between canine OS patients with high versus low PSMD14 and PSMA7 mRNA, alone and combined (GSE27217, log-rank (Mantel-Cox) test). (**B**) Levels of PSMD14 in primary tumour-derived OS cell lines: HOS, Abrams (Ab), OVC-cOSA-75 (75), OVC-cOSA-78 (78), secondary tumour-derived OS cell lines: OVC-cOSA-31 (31), D17, K7M2, and non-malignant mesenchymal stromal cells (MSC) and Madin-Darby canine kidney (MDCK) cells. (**C**) MDCK, MSC, D17 and OVC-cOSA-31 cells were treated with capzimin at 0.001, 0.01, 0.1, 1, 10 μM for 24 or 48 h and incubated with resazurin for 8 h before reading the absorbance at 570/600 nm (five technical replicates per treatment). IC_50_ curves depict normalized % viability, mean ± SD from *n* = 3 independent experiments. The IC_50_ was determined using a four-parameter variable slope model using Prism 9 software. (**D**) Capzimin (CZM) treatment resulted in a slight increase in HIF1α levels with treatment. Representative HIF1α immunoblot after D17 and OVC-cOSA-31 (31) cells were subjected to DMSO (−) or their respective CZM IC_50_ treatment (+) for 24 h (D17 = 205 nM, 31 = 140 nM) or 48 h (D17 = 400 nM, 31 = 280 nM). (**E**) CZM treatment led to an increase in ubiquitinated proteins 6 h post treatment. D17 and OVC-cOSA cells were treated with DMSO or CZM (D17 = 400 nM, 31 = 280 nM) for 6 h before lysis and adsorption with ubiquilin 1 tandem UBA (TUBE2) beads. Enriched ubiquitinated proteins were detected using an anti-ubiquitin antibody.

To determine if the proteins that were identified in our proteomic analysis (Figure 2B,D) are clinically relevant in OS patients, we investigated whether their mRNA counterparts associated with the development of metastasis, disease free interval, overall survival, and alkaline phosphatase levels in publicly available mRNA data sets. Very few significant associations were found within the six evaluated datasets, two canine and four human, and clinical outcomes (see Table S1 for a summary). However, there were noteworthy associations between PSMA7 mRNA levels and overall survival, and between PSMD14 mRNA levels and metastasis and overall survival. Human OS patients who developed metastasis in 5 years had higher levels of PSMD14 mRNA than those that did not, while canine OS patients with high levels of PSMD14 mRNA had shorter overall survival. The latter was also true when PSMA7 was considered alone and when combined with PSMD14 (Figure 3A). PSMD14 is part of the 19S regulatory subunit of the proteasome and is responsible for removing the ubiquitin tagged off ubiquitinated proteins [33,34,35]. Given this association in patient datasets, the recent interest in the ubiquitin-proteasome system in OS [36,37,38], and the recent discovery and thorough characterization of capzimin, a small molecule inhibitor that targets PSMD14 [39], we decided to further investigate PSMD14′s potential as a therapeutic target for OS in vitro. Upon evaluating PSMD14 in various primary and secondary derived OS cell lines of canine, mouse, and human origin by immunoblotting, we found that the levels of PSMD14 were quite uniform (Figure 3B). We chose to focus on secondary tumour-derived cell lines, D17 and OVC-cOSA-31, given that metastatic disease is the most clinically relevant in OS patients.

### 2.4. OS Cell Lines Are Sensitive to Capzimin Treatment and Demonstrate Increased Ubiquitinated Products following Treatment

To determine if PSMD14 would be a viable clinical target, we characterized the impact of capzimin, a selective inhibitor of PSMD14, on the viability of two metastatic canine OS cells, D17, OVC-cOSA-31 and two non-malignant cell lines, MDCK (Madin-Darby canine kidney) and canine MSC (stromal-derived mesenchymal stem cells). Cell lines were subjected to five doses of capzimin (0.001, 0.01, 0.1, 1, 10 μM) or DMSO for 24 or 48 h and the IC_50_ was determined (Figure 3C). The IC_50_ was relatively similar across all four cell lines but slightly higher for MSC, D17 and OVC-cOSA-31 at 48 h compared to 24 h (~200–500 nM at 24 h, and ~300–600 nM at 48 h for all cell lines). We next explored capzimin’s ability to cause the accumulation of HIF1α, a known proteasome target and ubiquinated protein. Capzimin exposure for 24 or 48 h at each cell line’s respective IC_50_ led to a slight increase in HIF1α (Figure 3D). The increase in ubiquitinated proteins could be seen as early as 6 h upon treatment with 400 nM capzimin for D17 and 280 nM for OVC-cOSA-31 (Figure 3E).

### 2.5. Capzimin Induces Apoptosis in a Dose-Dependent Manner in Canine OS Cells

**Figure 4:**
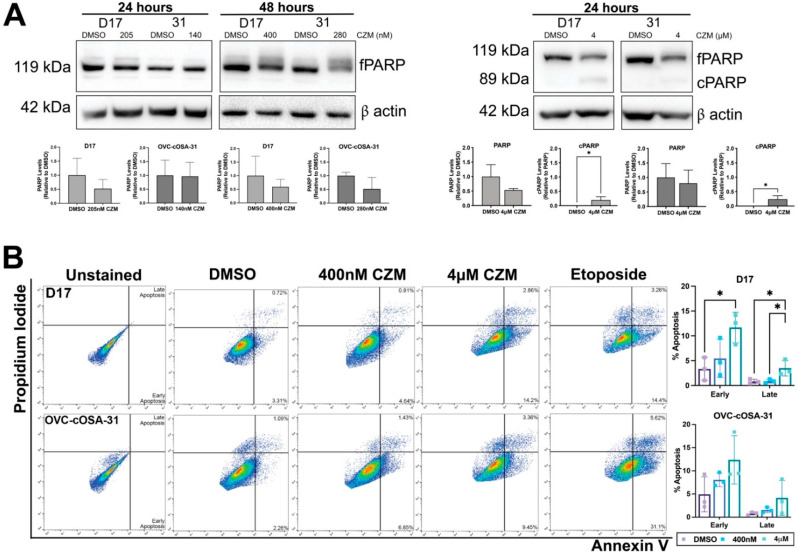
Capzimin induces apoptosis in a dose-dependent manner in canine OS cells. (**A**) CZM exposure at the calculated IC_50_ dose leads to a reduction in full length PARP (fPARP), but an associated increase in the cleaved version of PARP (cPARP) was not observed at either 24 or 48 h (left). However, when cells were treated with the 4 μM dose of CZM, the associated increase in cPARP was more evident (right). Immunoblots are from one representative experiment. fPARP levels were normalized to β actin (loading control) and compared to the DMSO control group to determine relative differences. cPARP levels were first normalized to β actin and are depicted as changes relative to normalized fPARP levels. Densitometry graph shows mean ± SD from *n* = 3 independent experiments; * indicates *p* < 0.05 as determined by an unpaired, two-tailed *t*-test. (**B**) Annexin V/Propidium Iodide flow cytometry analysis of OS cells treated with a low and high dose of CZM. A high dose of CZM significantly increases the percentage of early and late apoptotic D17 cells. Increases were seen in both cell lines when treated with the 400 nM of CZM, but these differences were not statistically significant. Graphs depict mean ± SD from *n* = 3 independent experiments; * indicates *p* < 0.05 as determined by a one-way ANOVA and post-hoc Tukey’s multiple comparison’s test.

Capzimin has previously been shown to induce cell cycle arrest, apoptosis, and DNA damage in the NCI-60 panel of cell lines [39]; however, none of these cell lines are of sarcoma origin. To evaluate capzimin’s ability to inhibit pro-tumourigenic traits, we exposed D17 and OVC-cOSA-31 cells to capzimin and assessed the levels of the apoptotic protein poly (ADP-ribose) polymerase (PARP). Although full length PARP (fPARP) levels decreased with capzimin treatment at the IC_50_, the concomitant increase in cleaved PARP (cPARP) was not observed at 24 or 48 h (Figure 4A, left). To determine if a higher dose was needed to induce apoptosis, the cells were exposed to a 4 μM dose capzimin, a concentration closer to the IC_50_ previously reported in human cancer cell lines [39]. Both canine OS cell lines demonstrated an increase in cPARP levels with the 4 μM exposure (Figure 4A, right). We further validated these results by performing flow cytometry analysis on apoptotic markers and observed a significant increase in the number of early and late apoptotic cells when D17 cells were treated with the 4 μM dose. Although there were increases observed in the number of apoptotic cells with OVC-cOSA-31, these differences were not found to be statistically significant (Figure 4B). These data suggest that high doses of capzimin are needed to generate a significant cytotoxic effect.

### 2.6. Capzimin Reduces Clonogenic Survival, Cell Growth, and Migration

**Figure 5:**
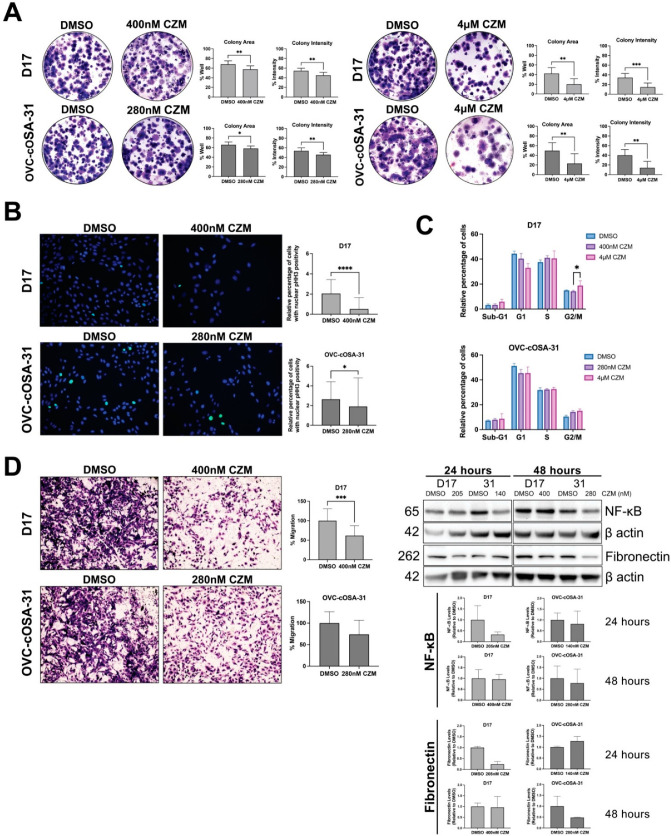
Capzimin reduces clonogenic survival, cell growth, and migration. (**A**) CZM reduces the clonogenic survival in both D17 and OVC-cOSA-31 cell lines at their respective IC_50_ dose (left) and the 4 μM dose (right). Colony area is defined as the area of the well occupied by cells, while colony intensity is defined as the pixel intensity of the colony. Graph depicts mean ± SD from *n* = 3 independent experiments, each experiment was seeded in 3 technical triplicates; * indicates *p* < 0.05, ** indicates *p* < 0.01, *** indicates *p* < 0.001 as determined by the Mann-Whitney test. (**B**) CZM reduces the percentage of mitotically active cells. Immunolabelling and quantification of DMSO or CZM-treated cells show differences in the nuclear levels of mitotic marker phosphorylated histone-H3 (pHH3). Graphs depict the relative nuclear positivity of pHH3 from *n* = 4 experiments, 5 images were evaluated per experiment; * indicates *p* < 0.05, **** indicates *p* < 0.0001. (**C**) CZM induces a G2/M arrest in D17 cells upon 4 μM treatment for 24 h. Graph depicts mean ± SD from *n* = 3 independent experiments (**D**) CZM decreases migration (left) and the expression of migration-associated markers (right). Images of a transwell insert stained with crystal violet 24 h post CZM or DMSO treatment at 10X magnification. Graphs show the percentage of migrated cells as determined after extracting stained transwell inserts with 10% acetic acid and measuring the absorbance at 590nm. The extracted dye was blank corrected to a media only stained insert and normalized to the DMSO control from *n* = 4 experiments, 2 technical replicates per treatment group; *** indicates *p* < 0.001. Representative immunoblots showing the impact of CZM treatment on NF-κB and fibronectin expression from one representative experiment. Protein levels were normalized to β actin (loading control) and compared to the DMSO control group to determine relative differences. Densitometry graph shows mean ± SD from *n* = 2 independent experiments.

To evaluate capzimin’s ability to inhibit colony formation, cells were treated with capzimin or DMSO, seeded at low density, and allowed to form colonies over a two-week period. Upon capzimin treatment, there was a marked reduction in the number and density of colonies formed in both cell lines when exposed to their IC_50_ or the 4 μM dose (Figure 5A). Given the proteasome’s ability to modulate the levels of cell cycle mediators [40], we next characterized capzimin’s impact on cell growth by evaluating the expression of the mitotic marker phosphorylated histone H3 (pHH3) and analyzing capzimin’s ability to influence the cell cycle. Capzimin significantly reduced the number of mitotically active cells at both the IC_50_ and 4 μM doses (Figure 5B). However, an effect on the cell cycle in the form of a G2/M arrest was only significant in D17 cells at the higher dose (Figure 5C), while a non-significant G2/M arrest was observed in OVC-cOSA-31 cells at both doses tested. Lastly, given the association between PSMD14 and metastasis, and the role of PSMD14 in mediating cell migration [41], we determined capzimin’s impact on migration using a transwell assay. Capzimin was found to significantly reduce migration in the D17 cell line, and with borderline significance in the OVC-cOSA-31 cell line (*p* = 0.0751) (Figure 5D, left). Capzimin’s ability to mediate migration could be explained by its ability to modulate the levels of migration-associated markers. Capzimin was found to decrease the levels p65 NF-κB/RelA and fibronectin in both cell lines, with effects observed on D17 and OVC-cOSA-31 cells at 24 h and 48 h, respectively (Figure 5D, right).

### 2.7. Capzimin Reduces Viability Compared to Doxorubicin Alone at Low Doses

**Figure 6:**
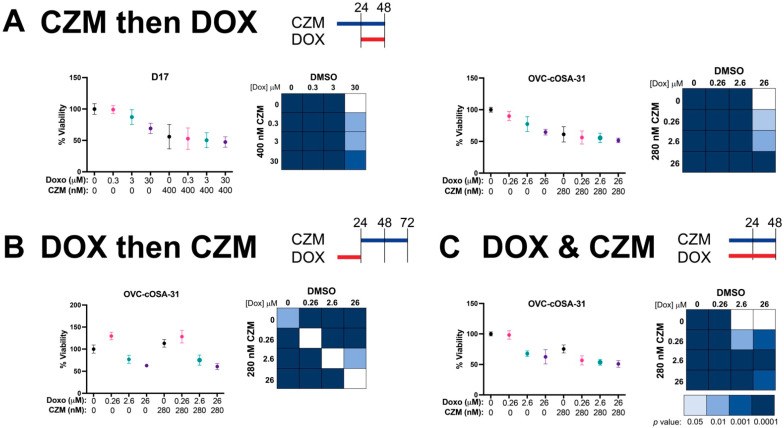
Capzimin sensitizes OS cells to doxorubicin (Dox) treatment. D17 or OVC-cOSA-31 cells were treated with subjected to three different conditions to evaluate CZM’s efficacy combined with Dox. (**A**) In the CZM and then Dox conditions, cells were treated with DMSO or their respective CZM for 24 h and then subjected to their IC_50_ dose of doxorubicin (30 μM for D17 and 26 μM for OVC-cOSA-31) or 10-fold or 100-fold lower concentrations for an additional 24 h. (**B**) In the Dox and then CZM condition, cells were treated with Dox for 24 h, after which, the media was removed and media with DMSO or CZM was replaced for 48 h. (**C**) In the Dox & CZM condition, cells were subjected to co-treatment of both compounds for 48 h. After each experimental time course, viability was measured by incubating with resazurin for 8 h and measuring absorbance at 570/600 nm (six technical replicates per treatment). Graphs depict normalized % viability, mean ± SD from *n* = 3 independent experiments, except for part C, where *n* = 2 independent experiments. Chart depicts select statistically significant differences as determined by a two-way ANOVA with a post-hoc Tukey’s multiple comparison test. Non-filled squares indicate no significant differences were observed.

As capzimin was unable to induce significant apoptosis at its IC_50_ as a single agent, and proteasome inhibitors have been previously shown to work synergistically with other chemotherapies [36,42], we determined its efficacy when combined with doxorubicin, a standard of care chemotherapy for both canine and human OS [43,44]. D17 and/or OVC-cOSA-31 cells were subjected to three different conditions: (1) treatment with capzimin and then doxorubicin; (2) doxorubicin and then capzimin, or (3) simultaneous treatment with doxorubicin and capzimin (Figure 6). In terms of the doxorubicin treatment, we used an IC_50_ previously calculated by our group (30 μM for D17, 26 μM for OVC-cOSA-31) [45], as well as 10-fold lower, or 100-fold lower concentrations. We chose these concentrations as the plasma concentration for doxorubicin in dogs is estimated to peak at ~3 μM [36].Viability assessment in both D17 and OVC-cOSA-31 suggests that sequential treatment with capzimin and doxorubicin (condition 1) resulted in reduced viability compared to doxorubicin alone, particularly at lower concentrations of doxorubicin (i.e., <IC_50_). However, none of the combination treatments were significantly different to capzimin treatment alone (Figure 6A). When considering sequential treatment of doxorubicin then capzimin (condition 2), capzimin ’s ability to reduce viability, either alone or combined with doxorubicin, was blunted (Figure 6B and Figure S1 for OVC-cOSA-31 and D17, respectively). Lastly, the simultaneous treatment of DOX and capzimin (condition 3, OVC-cOSA-31) yielded similar trends as in condition 1, as capzimin further reduced cell viability at lower concentrations of doxorubicin (Figure 6C).

### 2.8. Capzimin Reduces Viability of Metastatic Human OS Cells in 3D Conditions Resembling Secondary Sites

**Figure 7:**
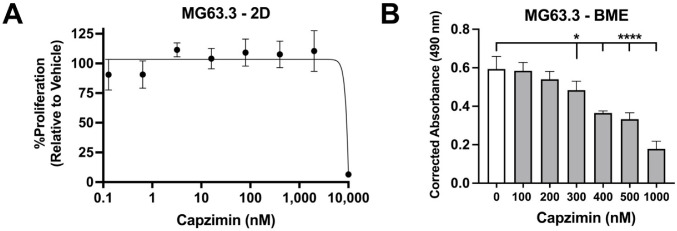
Human OS cells are more sensitive to capzimin when cultured under 3D conditions. (**A**) CZM was unable to blunt proliferation up to a 2 μM dose in MG63.3 cells treated in 2D conditions for 6 days. Graph depicts the relative absorbance values at 490 nm and normalized to the DMSO (vehicle) control and represents the mean ± SD from *n* = 3 independent experiments. (**B**) CZM significantly decreased growth under 3D conditions. MG63.3 cells were cultured in a 3D Basement Membrane Extract (BME) system and subjected to various doses of CZM treatment for 6 days. Growth was significantly decreased by CZM at 300, 400, 500 and 1 μM concentrations (* *p* < 0.05 and **** *p* < 0.0001). Graph depicts corrected absorbance values at 490 nm with BME + media alone used to subtract background. The results are representative of one of 2 experiments, each with 4 technical replicates.

Given the similarities in clinical presentation and molecular alterations between canine and human OS, canines have been highly regarded as a relevant, naturally occurring translational model for the human disease [1,46]. To supplement the clinical association we saw in published human datasets (Figure 3A), and determine capzimin’s effect on human OS cells, we treated the highly metastatic MG63.3 cell line with capzimin under standard 2D vs. 3D culture conditions. The 3D culture conditions aim to mimic disseminated tumour cells and metastatic outgrowth [47]. Interestingly, capzimin didn’t have an impact on cell number when cells were cultured in 2D conditions, even at a high dose of 2 μM (Figure 7A). However, when MG63.3 cells were cultured under 3D conditions there was an evident reduction in cell number upon exposure to as low as 300 nM capzimin (Figure 7B). This data suggests that capzimin has the ability to inhibit the metastatic outgrowth of human OS cells.

## 3. Discussion

EVs have been proven to be useful biological tools that aid in biomarker discovery and expand our understanding of cancer biology [32]. Unfortunately, EV research in the field of OS has been predominately limited to in vitro studies with cell lines. These in vitro models have limited clinical relevance as they rely on available cell lines and their behaviour in culture. To date, only one study has profiled EVs from human OS tumour explants, but this study, unfortunately, lacked non-malignant bone controls for comparison [32].

Herein, we developed a pipeline that allowed for the successful isolation of EVs from malignant and non-malignant bone tissue explants obtained from eight canine appendicular OS patients. By exploring the proteome of the isolated vesicle preparations, we found an interesting protein signature associated with the OS-derived vesicles. This signature related to protein metabolism, with several structural components of the ribosome upregulated (Figure 2B,C). Specifically, we found an upregulation of proteins associated with the 60S ribosome (60S acidic ribosomal protein P0, 60S ribosomal protein L12, L18) and 40S ribosome (40S ribosomal protein S2, S3, S3a, S27, S4), as well as several eukaryotic initiation factors (eIF3L, eIF3F, eIF3H, eIF2α). These findings are in line with what has been previously described in cancer biology as dysregulated protein synthesis and is considered a common aberrant feature of cancer cells [48]. In OS specifically, disseminated cells have been shown to increase mRNA translation to enhance metastatic colonization of the lung [49]. By boosting their protein translation machinery, OS cells may have the ability to increase translation capacity, especially during periods of stress [50].

Given the important role of protein translation in cancer, it is not surprising that it has been explored as a potential anticancer therapy. The most notable targets include factors involved in the eIF4 complex, a crucial protein complex in recruiting mRNA to the ribosome [48]. Aside from directly targeting eIF4 proteins, one strategy involves targeting upstream mediators such as the phosphoinositide 3-kinase/mammalian target of rapamycin (PI3K/mTOR) pathway, a pathway that is also frequently overactive in OS [51]. Upon exposing metastatic OS cells to the mTOR inhibitor rapamycin during lung colonization in a mouse model, there was a successful inhibition of overall translational output and an associated reduced metastatic burden and prolonged survival [49]. Despite the excitement derived from these preclinical studies, a recent randomized trial involving 324 dogs found there was no survival benefit when the mTOR inhibitor, sirolimus, was added to the standard of care therapy [52]. Based on this, we decided to explore other potentially targetable molecules identified by our proteomic analysis which showed clinical relevance.

We directed our focus to the deubiquitinating enzyme PSMD14 (26S proteasome non-ATPase regulatory subunit 14) also referred to as Rpn11 and POH1, given its relevance in published human and canine datasets (Figure 3A) and the availability of a well-characterized specific inhibitor at the time we initiated our studies. PSMD14 is located within the 19S regulatory particle of the proteasome, which, together with the 20S proteolytic core particle (CP), make up the proteasome. The core particle consists of both α and β rings and each β ring contains three sites to facilitate protein degradation: chymotrypsin-like (β5), trypsin-like (β2) and caspase-like (β1) [53]. The regulatory particle contains both base and lid subcomplexes: the base contains six ATPases (Rpt1-Rpt6) and four non-ATPase subunits (Rpn 1, 2, 10, and 13), while the lid contains nine non-ATPase subunits (Rpn 3, 5–9, 11, 12 and 15) [33,34]. PSMD14/Rpn11 is a metalloprotease which deubiquitinates poly-ubiquitinated proteins prior to their eventual degradation by the core particle [33,34,35]. Normal proteasome function is crucial for cell homeostasis by ensuring the timely degradation of cell cycle mediators and the degradation of misfolded proteins [53]. Several studies have demonstrated that cancer cells have a particularly heightened dependence on proteasomes, given their increased expression of mutated proteins and excessive protein synthesis due to aneuploidy [54]. This suggests that cancer cells are particularly sensitive to proteasome inhibition which can lead to fatal consequences [55].

To date, several proteasome inhibitors have been developed which mostly target the catalytic core of the proteasome. These include the “omib” series of proteasome inhibitors for the treatment of multiple myeloma: bortezomib (first generation, FDA approved for clinical use in 2003) [56], carfilzomib (second generation, FDA approved in 2012) [57], and ixazomib (second generation, FDA approved in 2015) [58]. Although there has been some preclinical and clinical success with these inhibitors in multiple myeloma, their activity in solid tumours has been disappointing [59]. Furthermore, multiple myeloma patients have been shown to develop rapid resistance to proteasome inhibitors, emphasizing the need to find new ways to target the proteasome [60]. To this end, we explored capzimin which is a selective inhibitor of PSMD14/Rpn11 with demonstrated activity in epithelial malignancies [39]. We found that two metastatic canine OS cells, D17 and OVC-cOSA-31, were sensitive to capzimin and increased the levels of ubiquitinated proteins as well as the known target, HIF1α, with treatment (Figure 3C,D). Previous work in human and canine OS cell lines found that proteasome inhibitors can reduce cell viability and cause apoptosis [39,41]. A similar effect was observed in this study when D17 and OVC-cOSA-31 were treated with their respective IC_50_ at 24 and 48 h (Figure 4A, left); however, a high dose (4 μM) of capzimin was needed to produce significant cytotoxic effects (Figure 4A, right and Figure 4B). This result could be explained when considering the kinetics of proteasome inhibition and cell death. Deshaies (2014) predicted that the cell’s proteasome needs to be inhibited by a certain percentage (a minimum of ~60%) to commit to apoptosis [54]. A lower dose may be unable to inhibit the proteasome to this degree and cells have also been shown to increase the expression of proteasome subunits upon proteasome inhibition [61].

Aside from exploring capzimin’s ability to induce apoptosis, we also explored capzimin’s influence on clonogenic survival and proliferation as cell cycle mediators are targets of the proteasome. Proteasome inhibitors have been shown to stabilize the levels of p53, a mediator of the G1/S and G2/M arrest, as well as the CDC25 proteins which are phosphatases that regulate the activity of certain cyclin/cyclin dependent kinase complexes [43,58]. The resultant stabilization of these molecules can alter the cell’s ability to transition through difference phases of the cell cycle and instead cause a growth arrest. Upon assessing proliferation using the clonogenic survival and quantifying the levels of pHH3, a marker of mitosis, we found that capzimin was able to significantly blunt clonogenic survival at low (280 and 400 nM) and high (4 μM) doses of capzimin and decrease the levels of pHH3 (Figure 5A,B). These results are in line with our cell cycle analysis as D17 cells exhibited a G2/M arrest upon capzimin treatment, but only at the high dose of 4 μM (Figure 5C). When we extended this analysis to the human OS cell line MG63.3, we interestingly found that capzimin doses ≤2 μM did not have an impact on proliferation when the cells were cultured under 2D conditions. However, when using a 3D culture previously shown to mimic outgrowth of disseminated cells [47], capzimin was shown to significantly blunt proliferation at doses as low as 300 nM (Figure 7). This demonstrates that capzimin might negatively impact the metastatic endurance of disseminated tumour cells and prevent their outgrowth and eventual colonization. Interestingly, capzimin might also interfere with earlier steps of the metastatic cascade, as it reduced cell migration in both D17 and OVC-cOSA-31 (Figure 5D, significant for D17), as well as NF-κB and fibronectin expression, although with different kinetics for each cell line (Figure 5D, right). NF-κB has been demonstrated to be important for OS metastasis [62], and its activity is regulated by inhibitory kappa B (IκB). IκB bound to NF-kB prevents its nuclear translocation and downstream transcription of target genes [63]. IκB is a target of the proteasome, and proteasome inhibition may prevent its degradation and thus modulate NF-κB [64]. Fibronectin has also been demonstrated to be important for OS metastasis as OS stem cells, which are hypothesized to contribute to metastases development, undergo a fibrogenic reprogramming resulting in fibronectin deposition. This allows for the eventual metastatic outgrowth of disseminated OS cells [65]. A previous study also found that proteasome inhibition could decrease fibronectin levels [66], further evidencing the negative impact of proteasome inhibition on various pro-tumour molecules.

To date, a few preclinical studies have shown that proteasome inhibitors may be effective in combination therapies against OS. For instance, bortezomib and carfilzomib are synergistic with histone deacetylase inhibitors, panobiostat and romidespin, in human in vitro and ex vivo models of OS [67], while combining bortezomib with doxorubicin and carboplatin induces more cytotoxic effects that either drug alone in canine OS cells [36]. In an OS xenograft model, the proteasome inhibitor MG132 combined with cisplatin proved to be a more useful treatment combination compared to each agent alone [42]. We decided to explore this possibility with capzimin based on the documented role of PSMD14/Rpn11 in repair of double strand DNA breaks [68], and the capacity of doxorubicin to cause double strand breaks at promoter sites [69]. Based on these precedents, we exposed D17 and/or OVC-cOSA-31 to either: (1) sequential treatment of capzimin at the IC_50_, and doxorubicin at three different doses 24 h later; (2) sequential treatment of doxorubicin at three different doses and then capzimin at the IC_50_, or (3) simultaneous treatment of doxorubicin and capzimin (Figure 6). Sequential treatment of capzimin and doxorubicin (Figure 6A) and simultaneous treatment of doxorubicin and capzimin (Figure 6C) resulted in a significant reduction in the viability of D17 and OVC-cOSA-31 when compared to doxorubicin alone for almost all doses of doxorubicin tested. However, since no significant differences were observed between capzimin alone and capzimin and doxorubicin at any of the concentrations tested for both cell lines, we speculate that the effects seen in the combination treatment are the effects of capzimin. Therefore, the drug combination does not further enhance the effect of capzimin alone. Interestingly, this was not seen in the sequential treatment of doxorubicin and then capzimin condition (Figure 6B and Figure S1). Capzimin did not significantly reduce the viability of D17 and OVC-cOSA-31 when added alone or when compared to doxorubicin alone for any of the combinations tested. Since we kept the degree of cell confluency constant at the beginning of the experiment, and the time (48 h) and dose of capzimin constant for all experiments, we hypothesize that this could be a result of contact inhibition. Capzimin-only treated cells were not subjected to any treatment for 24 h after the start of the experiment and thus continued to divide, resulting in a near-confluent well by the time treatment started. This suggests that capzimin may only be effective when cells are dividing.

It should also be noted that all treatments were conducted in the presence of 10% FBS and in media containing high levels of glucose. It is possible that reducing serum and glucose concentration might mimic better a stressful microenvironment and could possibly result in better effects of drug combinations. This would be in line with what has been previously described in the literature and would be likely attributed to how serum deprivation and hypoxic conditions can decrease topoisomerase IIα expression [70]. Topoisomerase IIα catalyzes double strand breaks in DNA to prevent supercoiling during replication [71]. Anti-cancer therapies, such as etoposide and doxorubicin, can bind and stabilize the cleavage complex generated by the toposiomerase IIα reaction. An accumulation of these complexes may ultimately lead to cell death [72,73,74]. By decreasing topoisomerase IIα expression levels, tumours can decrease the number of cleavage complexes formed and thus become resistant to such therapies. Interestingly, a study found that serum deprivation and hypoxic conditions can decrease the levels of topoismerase IIα, but this was negated with proteasome inhibition [70]. As our experiment was completed under full serum conditions, it is possible that topoisomerase IIα was present at adequate levels to execute its function.

Overall, our results show that OS-derived EVs have a protein signature related to protein metabolism, and capzimin has pro-apoptotic and anti-proliferative effects in OS cell lines. A major limitation of this study was its small sample size. Due to the low number of samples (*n* = 12) with adequate protein concentration for proteomic characterization (possibility reflecting insufficient quality of EV preparations), our proteomics analysis was suboptimal. Despite these shortcomings, we are encouraged by the results of this pilot study and believe this pipeline should be extended to larger cohorts to account for patient variability. Our data strongly suggest that the identification of the protein cargo of EVs released by tumour explants has the potential to enhance our collective understanding of OS biology and to identify effective prognostic and therapeutic targets. Future studies will need to validate the specificity of our identified protein signature as previous studies that profiled cancer EV proteomes have also found ribosomal proteins to be enriched in their dataset [30]. Protein translational machinery has notably been found in ectosomes compared to exosomes, which is likely the predominate type of vesicle in our preparations based on our size analysis [75]. However, it is possible that this signature is still unique to OS, as a study that profiled OS tumour tissue found an upregulation of 60S acidic ribosomal protein P0, proteasome subunits β type-4 (PSMB4) and β type-6 (PSMB6), and a signature relating to unfolded protein response which is targetable by proteasome inhibitors [76]. Furthermore, six of the proteins we identified as upregulated in the OS EV proteome were previously found to associate with metastatic disease, recurrence/disease free interval, or overall survival in human OS, these include: RPS3 [77], RPS9 [78], VCP [79], BUB3 [80], PHGDH [81] and endostatin, which is one of the two cleavage products of Col18A1 [82]. This gives us more confidence that our research platform is a feasible strategy to find protein signatures of clinical value in OS. Future studies will also need to determine how capzimin fairs in an in vivo setting, especially since previous in vivo studies with proteasome inhibitors have produced mix results [37,83,84,85]. It would also be interesting to compare capzimin to other published PSMD14 inhibitors, such as thiolutin [41] and *O*-phenanthroline [86], and determine their suitability for OS treatment.

We also recognize that our EV profiling was completed on primary OS tissue, and it is unclear if this signature is relatable to the metastatic lesion, given tumour heterogeneity. The metastatic tumour (lung) EV proteome may arguably be an even more important signature to unveil, especially since metastatic disease is the predominant cause of death in OS patients due to the current lack of effective anti-metastatic therapies. An extension of our developed pipeline is to characterize the EV proteome from lung metastases-derived explants. Leveraging the canine model is absolutely crucial in this regard, as we are very privileged to count on the support of dog owners that provide consent to recover and perform analysis in tissue specimens postmortem.

Lastly, although the focus of this study was to use tumour explants, another noteworthy advantage of EVs is their presence in biofluids. Previous estimates suggest that EVs are highly abundant in the blood, with quantities close to 10^10^ EVs/1 mL of plasma [87]. It is unclear if the signature that we have discovered in this study will also be found in the plasma/sera of canine or human OS patients, as a previous large-scale study that compared tissue explant EV signatures and plasma EV signatures found that they were not entirely congruent [32]. This is likely due to plasma/sera-EVs being reflective of one’s entire physiology, while the tumour explant is a more direct tumour source. Nonetheless, EV profiling in blood samples (liquid biopsies) have several advantages to traditional tumour biopsies, e.g., they are less invasive and allow for serial monitoring of a patient over time [88]. To the best of our knowledge, only two studies have explored the use of EVs as liquid biopsies in canine OS to date [89,90]. Future studies should explore this avenue as canine OS is lacking reliable pharmacodynamic and prognostic biomarkers (as discussed in our recent review [91]).

In summary, this study is a proof-of-principle that our pipeline can: (1) isolate EVs from OS tissue explants, (2) identify clinically relevant protein signatures, and (3) identify viable molecular targets. With this pipeline, we identified a protein signature related to protein metabolism and found that PSMD14/Rpn11 may be a viable target worthy of further exploration in OS.

## 4. Materials and Methods

### 4.1. Sample Acquisition and Explant Culture

Upon owner consent and immediately following limb amputation, OS samples were obtained using sterile biopsy tools, while normal bone samples were obtained using a sterilized ½, ¼, and 3/8-inch Milwaukee Diamond Plus hole saw drill bit from the opposite region of same bone (NB_same_). A phalangeal bone sample was also obtained as a second normal bone sample (NB_phal_) using a sterile scalpel blade. Sampling of the OS sample was guided by patient radiographs. All samples were placed in pre-weighed 50 mL conical tube containing sterile PBS. The tubes with the tissue samples were weighed after collection to determine the sample weight. Tissue samples were rinsed briefly with 70% ethanol and mechanically fragmented using sterile tools before placing into DMEM high glucose media supplemented 10% vesicle-depleted FBS (prepared using previously described methods [92]), 100 IU/mL Pencillin, 100 μg/mL Streptomycin, and 2.5 μg/mL of Amphotericin B. Tissue explants were incubated for 24 h under standard conditions, after which, the media was collected and spun at 400× *g* for 15 min at 4 °C twice to remove any debris. The media was transferred to a new 15 mL conical tube and stored at −80 °C until further use.

### 4.2. Vesicle Isolation

To isolate EVs from the explant media, the media was thawed at 4 °C overnight, and concentrated to ~500 μL using Amicon^®^ Ultra-15 Centrifugal Filters (Millipore Sigma, Burlington, MA, USA) and centrifuging at 4000× *g* for 15 min. The concentrated sample was then applied on top of a qEVoriginal SEC column (iZon Science, Medford, MA, USA) and 0.22 μM filtered sterile PBS was added to the column in 500 μL increments as the sample passed through the column. Twelve 500 μL fractions were immediately collected as they eluted from the column in microcentrifuge tubes. Fractions 7, 8, 9, 10, 11, and 12, which were expected to contain EVs, were further concentrated to ~150 μL volumes using Amicon*^®^* Ultra-0.5 mL centrifugal columns (Millipore Sigma, Burlington, MA, USA) and centrifuging at 14,000× *g* for 5 min. The flow through from the column was discarded, while the column was inverted to a new tube and spun at 1000 *g* for 3 min to recover the concentrated sample.

### 4.3. Vesicle Lysis

Isolated vesicle fractions were lysed by adding an equal volume of lysis buffer, consisting of 10× cell lysis buffer (Cell Signaling Technology, Danvers, MA, USA) diluted in a 1:5 dilution, 2 μg/mL aprotinin (Sigma Aldrich, St. Louis, MO, USA), 1% phosphatase inhibitor cocktail (Sigma Aldrich, St. Louis, MO, USA), 1 mM sodium orthovanadate (New England Biolabs, Ipswich, MA, USA) and 1 mM PMSF (Sigma Aldrich, St. Louis, MO, USA). Samples were incubated on ice for 30 mins and then centrifuged at 15,000× *g* at 4 °C for 20 min and the supernatant was stored at −80 °C until further use.

### 4.4. Dynamic Light Scattering

Fractions that demonstrated immunoreactivity for Flotillin-1 and/or CD63 were pooled in equal volumes and diluted in a 1:10 dilution with 0.22 μM sterile-filtered PBS in a disposable Malvern Panalytical 40 μL cuvette (Malvern Panalytical, Malvern, England) and subjected to dynamic light scattering using the Malvern Zetasizer Nano S. The following specifications were used: Dispersant = ICN PBS Tablets, Dispersant RI = 1.330, Viscosity (cP) = 0.8882, Material RI = 1.38, Material Absorption = 0.010. Each sample was read in three technical replicates.

### 4.5. Transmission Electron Micrscopy

Five microliters of a vesicle preparation were placed on an electron microscopy grid. Samples were fixed to the grid using paraformaldehyde and stained using uranyl acetate. After 2 minutes, samples were imaged using a FEI Tecnai G2 F20 microscope (FEI Company, Hillsboro, OR, USA).

### 4.6. Vesicle Protein Quantification

The protein concentration of lysed vesicle fractions was determined using the Pierce BCA Protein Assay Kit (Thermo Fisher Scientific, Waltham, MA, USA) as per manufacturer’s protocol. Briefly, 25 μL of each vesicle sample and protein standards were pipetted in duplicate in a 96 well plate. A 1:50 dilution of the working reagent was created and a 200 μL volume was added to each well. The plate was vigorously shaken for 30 s at 500 rpm and incubated at 37 °C for 2 h. Once the plate cooled, the absorbance was measured at 562 nm using a Synergy 2 Microplate Reader (BioTek, Winooski, VT, USA). Protein concentration was calculated by comparing the absorbance values to a standard curve generated with known protein standards.

### 4.7. Ultra High Performance Liquid Chromatography and Tandem Mass Spectrometry (UHPLC-MS/MS)

UHPLC-MS/MS was conducted by the McGill University Health Centre and Proteomics Platform. Samples with CD63 and/or flotillin expression and a minimum concentration of 2.9 μg total protein were sent to McGill University to be analyzed. An equal concentration of 2.9 μg of EV lysate was loaded onto the gel for each sample, followed by staining and de-staining. Gels were reduced with DTT, alkylated with iodoacetic acid, and digested with trypsin. The peptides were then re-solubilized in 0.1% aqueous formic acid and 2% acetonitrile. The sample was then loaded into an Acclaim Pepmap Easyspray analytical separation column in the Dionex Ultimate 3000 uHPLC. The samples were run at 220 nL/min with a gradient of 2–35% organic (0.1% formic acid in acetonitrile) over 3 h. The samples were then run through a Thermo Orbitrap Fusion Mass Spectrometer at a 120,000 resolution. The raw data collected were compared to the Mascot 2.3 database against canine sequences and analyzed using Scaffold Q+ (Proteome Software, Portland, OR, USA). Genes of interest were further analyzed using FunRich with the UniProt database and the Cytoscape Reactome FI application [93]. A student’s *t*-test was used to determine the differences between non-malignant and OS samples, based on a protein and peptide threshold of 95% and a minimum of two peptides unless otherwise indicated.

### 4.8. Associations of mRNA Expression of Identified Proteins with Clinical Outcome

Processed human (GSE14827, GSE21257, GSE32981, and GSE39058) and canine (GSE27217 and GSE63476) datasets with corresponding gene expression and clinical annotation files were downloaded from the Gene Expression Omnibus (GEO). Data was log2 transformed if needed, and in the case of microarrays with multiple probe sets mapping to a single gene, the probe set with the highest variance across samples was selected. To test the association of the mRNA for each specific gene with metastasis, expression values were compared using Welch’s *t*-test or ANOVA. For survival analysis, samples were split into high and low expression groups using the median as a bifurcation point and Kaplan-Meier curves were compared using the log-rank test. All graphical and statistical analyses were performed using Prism v9.0 software (GraphPad Software, San Diego, CA, USA) [94].

### 4.9. OS Cell Culture Conditions

Commercially available D17, MDCK, HOS, Abrams, Ontario Veterinary College-generated OVC-cOSA-31, OVC-cOSA-75 and OVC-cOSA-78, MG63.3 (obtained via MTA by Vuja De Science Inc., Natick, MA, USA), and canine stromal-derived MSC (a gift from Dr. Jonathan Lamarre, Dept. Biomedical Sciences, University of Guelph, Canada) were maintained in DMEM High Glucose media supplemented with 10% FBS, 100 IU/mL Penicillin, and 100 μg/mL Streptomycin under standard culture conditions.

### 4.10. Capzimin IC_50_ Dose Response

MDCK, MSC, D17 and OVC-cOSA-31 cells were seeded at a density of 7500 cells/100 μL media in 96 well plate in six technical replicates. Cells were allowed to adhere overnight and the next day, capzimin (Cat #SML1995; Sigma Aldrich, St. Louis, MO, USA) was added at 0.001, 0.01, 0.1, 1μM, or 10μM concentrations for 24 or 48 h. Post treatment, 10 μL of resazurin (R&D Systems) was added to each well and incubated for 8 h at 37 °C. Post incubation, the absorbance was determined at 570 nm and 600 nm using the Synergy 2 Microplate Reader (BioTek, Winooski, VT, USA). Cell viability was determined relative to the DMSO control and the IC_50_ was calculated using the variable slope (four parameters) model.

### 4.11. Capzimin Treatment & Lysate Collection for the Detection of Apoptosis

To evaluate the impact of capzimin on apoptosis, 1.0 × 10^6^ cells were seeded in 100 mm dishes and treated with the calculated IC_50_ or 4 μM of capzimin for 24 h or the calculated IC_50_ for 48 h. A volume equivalent of DMSO was used as a vehicle control. To lyse dead cells, the media of treated cells was collected and centrifuged at 800× *g* for 15 min at 4 °C, while adherent cells were incubated with lysis buffer and collected into a tube with a cell scraper. Following the centrifugation of dead cells, the media was aspirated and the pellet containing dead cells was lysed with lysis buffer and combined with the adherent cells. The lysate mixture was incubated on ice for 30 min and then centrifuged for 20 min at 15,000× *g* at 4 °C. The lysis buffer was prepared similarly to the one used for vesicle lysis, but a 1:10 dilution of the 10× lysis buffer was used instead.

### 4.12. Cell Lysate Protein Quantification

The protein concentration of collected cell lysates was determined using the DC protein assay (BioRad, Hercules, CA, USA). Briefly, 5 μL of each cell lysate and known protein standards were pipetted in duplicate in a 96 well plate. A 1:50 dilution of the Protein Assay Reagent A (BioRad, Hercules, CA, USA) and Protein Assay Reagent S (BioRad, Hercules, CA, USA) was created, and a 25 μL volume was added to each well. Two hundred microliters of Protein Reagent B (BioRad, Hercules, CA, USA) was added and the plate allowed to incubate for 10 minutes rocking at room temperature. After the incubation, the absorbance was measured at 630 nm using the Synergy 2 Microplate Reader (BioTek, Winooski, VT, USA). Protein concentration was calculated by comparing the absorbance values to a standard curve generated with known protein standards.

### 4.13. Immunoblotting of Vesicle Fractions and Capzimin Treated Cells

Twenty microliters of lysed vesicle preparations were used to determine the presence of CD63 and flotillin-1, 3 μg of lysed vesicle samples were used to characterize the expression of fibronectin, filamin A, stomatin and gelsolin, and 50 μg of cell lysates were used to detect PARP, PSMD14, HIF1α and β actin. Lysates were either resolved on 7.5, 10, or 15% polyacrylamide gels, depending on the size of the protein to be detected. After gel electrophoresis, gels were transferred to an activated 0.45 μm PVDF membrane under semi-dry conditions (for 15% gels, 17 V for 35 min) or wet transfer conditions (for 7.5% and 10% gels, 100 V for 2 h). Membranes were all blocked with 5% non-fat skim milk powder diluted in TBS + 0.1% Tween (TBST) for one hour at room temperature. Membranes were incubated with their respective antibody overnight at 4 °C with rocking. See Table S2 for a list of the antibodies used in this study. The following day, membranes were washed three times (10 min each) with TBST, and incubated with a 1:10,000 dilution of HRP conjugated goat anti-rabbit or HRP conjugated goat anti-mouse secondary antibodies (Sigma Aldrich, St. Louis, MO, USA) diluted in milk for 1 h rocking at room temperature. Membranes were washed three times (10 min each) with TBST, and Immobilon^®^ Forte Western HRP Substrate (Millipore Sigma, Burlington, MA, USA) was applied and imaged with ImageLab Software (BioRad, Hercules, CA, USA) [95].

### 4.14. Ubiqutinated Protein Enrichment

Two 100 mm dishes of D17 and OVC-cOSA-31 cells were seeded at a density of 1.0 × 10^6^ cells per dish. Cells were treated with their respective IC_50_ (400 nM for D17 and 280 nM for OVC-cOSA-31) for 6 h. Following the treatment, cells were lysed with lysis buffer, consisting of 10× cell lysis buffer (Cell Signaling Technology) diluted in a 1:10 dilution, 2 μg/mL aprotinin (Sigma Aldrich, St. Louis, MO, USA), 1% phosphatase inhibitor cocktail (Sigma Aldrich, St. Louis, MO, USA), 1 mM sodium orthovanadate (New England Biolabs, Ipswich, MA, USA), 1mM PMSF (Sigma Aldrich, St. Louis, MO, USA), 100 nM N-ethylmaleimide (Sigma Aldrich, St. Louis, MO, USA), and 100 nM iodoacetamide (Sigma Aldrich, St. Louis, MO, USA). Lysates were pooled and incubated on ice for 30 min and then centrifuged at 15,000× *g* at 4 °C for 20 min. Protein concentration was determined using the DC protein assay as mentioned above, and 1 mg of lysate was incubated with 25 μL of agarose ubiquilin 1 tandem UBA (TUBE2) beads (R&D Systems, Minneapolis, MN, USA) for 4 h rocking at 4 °C. Post incubation, the lysate-bead mixture was centrifuged at 3000× *g* for 5 min and the unbound lysate was aspirated. The agarose beads were washed with 500 μL PBS and centrifuged at 3000× *g* for 5 min. Upon removal of PBS, the beads were eluted with 2× SB and heated at 95 °C for 5 min. The eluent was subjected to immunoblotting using the protocol mentioned above.

### 4.15. Annexin-V FITC and Propidium Iodide (PI) Staining and Flow Cytometry

D17 and OVC-cOSA cells were seeded at a density of 0.8 × 10^6^ cells in a 60 mm dish overnight. The following day, cells were treated with either 400 nM or 4 μM capzimin, 50 μM etoposide (positive control), or DMSO for 24 h. Post treatment, spent media was collected and adherent cells were trypsinized, harvested and stained using the eBioscience Annexin V Apoptosis Detection Kit FITC (Thermo Fisher Scientific, Waltham, MA, USA). Dead and adherent cells were spun at 4000 × *g* for 5 min, washed with PBS and resuspended in 200 μL of 1× binding buffer. Cells were then stained with 5 μL Annexin V-FITC for 15 min and then 5 μL PI for 5 min at room temperature. Cells were then immediately flow sorted using BD Accuri C6 flow cytometer (BD Biosciences, Franklin Lakes, NJ, USA). A total of 50,000 events were measured per group and unstained (negative control) and fluorescent minus one etoposide-treated controls (positive controls) were used to determine the gating strategy and compensate for spillover between FITC (FL1) and PI (FL3). Data was analyzed using FlowJo v10.8 software (BD Biosciences, Franklin Lakes, NJ, USA) [96].

### 4.16. Clonogenic Survival Assay

D17 and OVC-cOSA-31 cells were seeded at density of 0.80 × 10^6^ cells in a 60 mm dish overnight. The next day, cells were treated with their respective IC50, 400 nM for D17, 280 nM for OVC-cOSA-31 for 48 h, or 4 μM for 24 h. Post treatment, cells were trypsinized and seeded at a density of 500 cells per well of a 6-well dish. Each treatment group was seeded in triplicate. Colonies were allowed to form over a two-week period and media was changed on day 7. After the two-week period, media was aspirated, and wells were washed with PBS before staining with crystal violet solution for 20 mins with rocking. Unbound dye was pipetted off and wells were thoroughly rinse with deionized water. Plates were allowed to dry and then were imaged and quantified using the Colony Area plugin on ImageJ. Three independent experiments were completed, and each experimental group was plated in triplicate.

### 4.17. Phosphorylated Histone-H3 (pHH3) Immunoblabelling and Quantification

D17 and OVC-cOSA-31 cells were seeded at density of 75,000 cells on 22 mm glass coverslips (Thermo Fisher Scientific, Waltham, MA, USA) and treated with 400 nM or 280 nM capzimin for 24 h, respectively. Post treatment, coverslips were fixed with 4% paraformalehyde diluted in PBS and immunolabelled for pHH3. Briefly, cells were permeabilized with 0.3% Triton-X for 15 min, washed with PBS and then blocked with 5% normal donkey serum diluted in PBS for 1 h at room temperature. Blocking was tipped off and coverslips were incubated with a 1:20,000 dilution of pHH3 (Abcam, ab5176) overnight at 4 °C in a humidified chamber. The following day, coverslips were washed with PBS, incubated with donkey anti-rabbit Alexa 488 antibody (Thermo Fisher Scientific, Waltham, MA, USA) for 45 min in the dark at room temperature. After incubation, coverslips were washed with PBS for 5 min and stained with 300 nM DAPI for 15 min, washed with PBS and coverslipped with Dako Mounting Medium (Agilent Technologies, Santa Clara, CA, USA). Coverslips were visualized with an epifluorescent microscope, and five random fields were imaged at 20× magnification per treatment group. DAPI stained cells were quantified using ImageJ [97], while pHH3-positive cells were manually quantified. The percentage of mitotically active cells was determined by dividing the number of pHH3 positive cells by the number of nuclei (DAPI) present in the field of the view and multiplying by 100. Percentages were averaged across all five images and four independent experiments.

### 4.18. Cell Cycle Analysis

D17 and OVC-cOSA cells were seeded at a density of 1 × 10^6^ cells in a 100 mm dish overnight. The following day, cells were treated with either DMSO, 400 nM, or 4 μM for 24 h. Post treatment, cells were trypsinized, harvested, and fixed in cold 70% ethanol for 1.5 h at 4 °C. Post fixation, cells were centrifuged at 400× *g* for 5 min, and the ethanol was removed. The cell pellet was washed twice with PBS and resuspended in 500 μL of FxCycle PI/RNAse Staining Solution (Thermo Fisher Scientific, Waltham, MA, USA) or PBS (unstained control) for 30 min at room temperature in the dark. Cells were then immediately flow sorted using a BD Accuri C6 flow cytometer (BD Biosciences, Franklin Lakes, NJ, USA). A total of 50,000 events were measured per group. Data was analyzed using the Watson (Pragmatic) model on FlowJo v10.8 software (BD Biosciences, Franklin Lakes, NJ, USA) [96].

### 4.19. Transwell Assay

D17 and OVC-cOSA-31 cells were seeded in Falcon^®^ 8 μm transwell inserts (Corning Inc., Corning, NY, USA) at a density of 2 × 10^4^ cells/200 μL of media and placed into a 24-well companion plate with complete culture media. Cells were allowed to adhere for 7–8 h and then treated with 400 nM (D17) or 280 nM (OVC-cOSA-31) capzimin diluted in serum starvation media (0.2% FBS) for 24 h. Media was removed from the insert, and they were stained with crystal violet for 20 mins with rocking. Inserts were rinsed to remove excess dye and allowed to dry overnight. Non-migrated cells were removed from the top of the insert with a cotton swab. To quantify the degree of cell migration, crystal violet stain was extracted from the insert with 10% acetic and the absorbance was measured at 590 nm using a spectrophotometer. All readings were blank corrected with an insert containing no cells. All experimental groups were seeded in duplicate and extracted dye readings were performed in duplicate.

### 4.20. Combination Treatment: Capzimin and Doxorubicin

D17 and OVC-cOSA-31 cells were seeded at a density of 7500 cells/100 μL in 96 well plate in six technical replicates. Cells were allowed to adhere overnight and the next day, subjected to one of three conditions. In condition 1, cells were subjected to capzimin and then doxorubicin. Capzimin was added at the appropriate concentration, 400 nM (D17) or 280 nM (OVC-cOSA-31), or an equivalent volume of DMSO treatment for 24 h. Twenty-four hours later, 50 μL of doxorubicin was added such that the final concentration was as follows: 0.3, 3, 30 μM (D17) or, 0.26, 2.6 or 26 μM (OVC-cOSA-31). The plate was incubated for an additional 24 h. In condition 2, cells were subjected to doxorubicin and then capzimin. Doxorubicin was added at similar concentrations as condition 1 for 24 h. Twenty-four hours later, the doxorubicin media was removed and replaced with media containing capzimin or DMSO. The plate was incubated for additional 48 h. In condition 3, cells were subjected to simultaneous treatment of capzimin + doxorubicin or DMSO + doxorubicin for 48 h using the doses above. Following each respective conditions’ incubation periods, 10 μL or 15 μL (10% of well volume) of resazurin (R&D Systems, Minneapolis, MN, USA) was added to each well and incubated for 8 h at 37 °C. Post incubation, the absorbance was determined at 570 nm and 600 nm using the Synergy 2 Microplate Reader (BioTek, Winooski, VT, USA). Cell viability was determined relative to the DMSO control.

### 4.21. Assessment of MG63.3 Cytotoxicity in 2D and 3D Culture 

MG63.3 cells were plated in 96 well plates at 1000 cells/well and incubated at 37 °C for 24 h. After 24 h, media was replaced with fresh media containing capzimin at the desired concentration and incubated at 37 °C for 6 days. After 6 days, MTT reagent (20 μL/well of 5mg/mL stock solution in sterile water; Sigma) was added to each well and the plate was incubated at 37 °C for 2 h. Media and MTT reagent was removed from all wells and DMSO (100 μL/well) was then added to each well to solubilize the formazan crystals. The resultant absorbance was measured at 490 nm using a plate reader (Molecular Devices, San Jose, CA, USA). For the 3D culture, Culturex^®^ PathClear, Reduced Growth Factor Basement Membrane Extract (BME; Biotechne, Minneapolis, MN, USA) was added (50 μL/well) to a 96 well plate and allowed to gel by incubating at 37 °C for ≥30 min. MG63.3 cells were harvested, washed with DMEM (no glucose, no FBS; Gibco, Waltham, MA, USA) and centrifuged at 1500× *g* for 5 min. The media was aspirated, and the remaining cell pellet was flicked for ~10 s to ensure disaggregation. MG63.3 cells were diluted in media containing capzimin and 2% BME, resuspended vigorously, and 150 μL of the cell suspension was added on top of the gelled 3D BME layer. Proliferation was determined in the DMSO control or capzimin treated wells 6 days post treatment through the addition of 20 μL MTS reagent (Promega), followed by incubation at 37 °C for 2 h and absorbance measurements at 490 nm via a plate reader (Molecular Devices, San Jose, CA, USA).

### 4.22. Statistical Analyses

All statistical analyses were performed using Prism v9.0 software (GraphPad Software, San Diego, CA, USA) [94], unless indicated otherwise. A *p* < 0.05 was considered statistically significant.

## Data Availability

Publicly available data sets used in this study can be accessed using the following links: GSE63476 (https://www-ncbi-nlm-nih-gov.ezproxy.u-pec.fr/geo/query/acc.cgi?acc=GSE63476 (accessed on 17 July 2019), GSE27217 (https://www-ncbi-nlm-nih-gov.ezproxy.u-pec.fr/geo/query/acc.cgi?acc=GSE27217 (accessed on 17 July 2019), GSE32981 (https://www.ncbi.nlm.nih.gov/geo/query/acc.cgi?acc=GSE32981 (accessed on 17 July 2019), GSE14827 (https://www-ncbi-nlm-nih-gov.ezproxy.u-pec.fr/geo/query/acc.cgi?acc=GSE14827 (accessed on 17 July 2019) GSE21257 (https://www.ncbi.nlm.nih.gov/geo/query/acc.cgi?acc=gse21257 (accessed on 17 July 2019)), GSE39058 (https://www.ncbi.nlm.nih.gov/geo/query/acc.cgi?acc=GSE39058 (accessed on 17 July 2019).

## Supplementary Materials

The following are available online at www.mdpi.com/article/10.3390/ijms23063256/s1.
