# Supplementary material for: Proteomic Assessment of Extracellular Vesicles from Canine Tissue Explants as a Pipeline to Identify Molecular Targets in Osteosarcoma: PSMD14/Rpn11 as a Proof of Principle
Source: Int J Mol Sci. 2022 Mar 17;23(6):3256. doi: 10.3390/ijms23063256 (PMC8953151; doi:10.3390/ijms23063256)
Supplement: Supplementary file 1 [file ijms-23-03256-s001.zip › Supplemental Information_EV Manuscript.pdf]

Table S1. Proteins elevated in OSA derived vesicles and their associations with metastasis, survival, and ALP levels in canine and human published datasets.

Dataset	Metastasis	Survival	ALP levels
**GSE63476 (Canine)**			RPLP0<br>RPS3<br>RPS9<br>RPL12<br>Ribosomal Combined<br>PSMA7<br>PSMD14
**GSE27217 (Canine)**	PSMD14<br>PSMA7<br>PSMD14 & PSMA7	**PSMD14***<br>**PSMA7***<br>**PSMD14 & PSMA7***	
**GSE32981 (Human)**	Ribosomal Combined<br>RAN<br>EIF3S6IP<br>RSP3<br>RSP9<br>RPL12<br>**RPLP0***<br>PSMD14		
**GSE14827 (Human)**	RPL0<br>Ribosomal Combined<br>PSMA7<br>PMSD14<br>PSMD14 & PSMA7		
**GSE21257 (Human)**	RPL0<br>PSMA7<br>**PSMD14***<br>PSMD14 & PSMA7	Ribosomal Combined<br>RPL90<br>RAN<br>E1F3S6IP<br>RPS3<br>RPL12<br>ST13<br>RPL0<br>PSMA7<br>PSMD14<br>PSMD14 & PSMA7	
**GSE39058 (Human)**		RPL0<br>RAN<br>EIF3L<br>RSP3<br>RSP9<br>**ST13***<br>RPL12<br>Ribosomal Combined<br>PSMD14	

* Denotes significant differences were observed; "Ribosomal Combined" includes RPLP0, RPS3, RPS9, RPS12

Table S2. List of antibodies used in this study.

Target	Vendor	Catalog Number	Dilution for Immunoblotting
CD63	Novus Biologicals	NBP2-42225	1:1000
Flotillin-1	BD Biosciences	610821	1:1000
Fibronectin	Novus Biologicals	NBP1-91258	1:1000
Filamin A	Sigma	MAB1678	1:1000
Stomatin	Abcam	ab166623	1:1000
Gelsolin	Abcam	ab11081	1:1000
PARP	Cell Signaling Technologies	9542	1:1000
β actin	Cell Signaling Technologies	4967	1:5000
PSMD14	Abcam	ab109123	1:1000
HIF1α	Novus Biologicals	NB100-105	1:1000
NF-κB	Cell Signaling Technologies	8242	1:1000
Ubiquitin	Enzo Life Sciences	ADI-SPA-200	1:1000
